# VoSeq: A Voucher and DNA Sequence Web Application

**DOI:** 10.1371/journal.pone.0039071

**Published:** 2012-06-12

**Authors:** Carlos Peña, Tobias Malm

**Affiliations:** 1 Department of Biology, University of Turku, Turku, Finland; 2 Department of Biology, University of Eastern Finland, Joensuu, Finland; J. Craig Venter Institute, United States of America

## Abstract

There is an ever growing number of molecular phylogenetic studies published, due to, in part, the advent of new techniques that allow cheap and quick DNA sequencing. Hence, the demand for relational databases with which to manage and annotate the amassing DNA sequences, genes, voucher specimens and associated biological data is increasing. In addition, a user-friendly interface is necessary for easy integration and management of the data stored in the database back-end. Available databases allow management of a wide variety of biological data. However, most database systems are not specifically constructed with the aim of being an organizational tool for researchers working in phylogenetic inference. We here report a new software facilitating easy management of voucher and sequence data, consisting of a relational database as back-end for a graphic user interface accessed via a web browser. The application, VoSeq, includes tools for creating molecular datasets of DNA or amino acid sequences ready to be used in commonly used phylogenetic software such as RAxML, TNT, MrBayes and PAUP, as well as for creating tables ready for publishing. It also has inbuilt BLAST capabilities against all DNA sequences stored in VoSeq as well as sequences in NCBI GenBank. By using mash-ups and calls to web services, VoSeq allows easy integration with public services such as Yahoo! Maps, Flickr, Encyclopedia of Life (EOL) and GBIF (by generating data-dumps that can be processed with GBIF's Integrated Publishing Toolkit).

## Introduction

The advent of molecular methods, such as DNA sequencing, has facilitated a rapid development of hypotheses for phylogenetic relationships among biological groups. The amount of DNA sequences that can be used in phylogenetic inference is growing at an increasingly fast pace. There are, as of now, more than 135 million sequences in GenBank [1] and 1.5 million DNA barcodes in BOLD [2]. Thanks to the development of new techniques and the possibility to outsource the processes involved, DNA sequencing is becoming cheaper and quicker. Thus, research groups might need the use of an efficient system in order to keep track of, manage and annotate their DNA sequences. In addition to adequate storage of DNA sequences, it would be advantageous if this system facilitates further processing of the data such as easy creation of datasets for phylogenetic analysis, quick creation of tables ready for publication and submission of sequences to public repositories (e.g. GenBank).

Relational databases permit organized storage of information that can be searched and retrieved quickly, depending on (and flexible to) the needs of users. However, relational databases such as MySQL need to be coupled with a software interface designed to act as a bridge between users and relational database for uploading, managing and retrieving e.g. DNA sequence and voucher specimen data.

Although there are several database systems available that handle biological and molecular data, they are conceived to be multipurpose systems [3]. Current biological databases handle a variety of information such as scientific names, current taxonomic classification, voucher images, geographic distribution maps, bibliographic references and so on. The Scratchpads project [4] is a social network portal for managing, sharing and publishing taxonomic information and voucher data online, but includes very limited capabilities for handling DNA sequence data (e.g. The Holometabola Insects Phylogenetic Database is based on Scratchpads [5]). The Lifedesks platform [6] hold taxonomic information and specimen data connected to voucher specimens in EOL, but there are no capabilities for storing and processing DNA sequences. The Mantis biological database manager allows storage of taxonomic and specimen data for museum collections [7]. These systems allow being configured so that they can used as private working platforms restricted to members of a research group.

The Barcode of Life Database (BOLD [8]) is both a public repository of DNA barcodes (and voucher data) and an online workbench module to collect and analyze DNA barcode sequences (a segment of the COI gene), but offers limited functionality for other gene markers as well as for dataset creation. Another commonly used database to store DNA sequences is GenBank, which is an excellent repository of data. However, GenBank is mainly used as a repository of sequences after they have been managed and analyzed during the research process. After the analysis of sequences is finished, the sequences are submitted to GenBank during the preparation of manuscripts. In this way, GenBank becomes a repository of end-products (sequences) of the research process.

Thus, there is a need for a database system that can be used to store and manage DNA sequences during the research process. This system should facilitate the aggregation of sequences into dataset files ready-to-run in common phylogenetic software. It should be able to handle the amount of data normally used during phylogenetic research: such as biological data from hundreds of voucher specimens and thousands of DNA sequences for a number of gene markers.

Here we present VoSeq, a user friendly database system designed for day to day use by researchers in phylogenetic inference to store and organize DNA sequences. VoSeq also stores complementary data such as voucher photos, collection data, taxonomic classification and collection locality maps. VoSeq can be downloaded and installed by on-screen instructions on a private computer, or installed on a shared server so that it can be used as a web application restricted to a research group or a network of collaborators. The main functionality lies in retrieving ready-to-run datasets for phylogenetic analyses for various software, complete with partition tables and analysis specifications, as well as MS Excel tables for work overview or publications. The platform of this database is taxon independent and can be used for all organisms. The source code is freely available under the GNU General Public License v2. Additionally, VoSeq has been designed to be cooperative with other biological web-based databases. For this, we included features of the so-called Web 2.0 [9] such as the Ajax protocol for user-friendliness, and exchange of information over the Internet based on SOAP and REST calls using the XML and JSON formats.

## Materials and Methods

VoSeq is written in the PHP scripting language to dynamically generate HTML and JavaScript code. All the information is stored in a MySQL relational database within several table trees. The database is designed to be used as a standalone application and accessed by using a standard web browser (e.g. Microsoft Internet Explorer, Mozilla Firefox or Google Chrome) provided that it is run on top of web server software (e.g. Apache [10]). If VoSeq is installed on a public server, it becomes a web server application, facilitating simultaneous collaboration between users from different geographic locations.

## Results and Discussion

### Design and implementation

After download, VoSeq is installed by following the on-screen instructions to complete the configuration process. VoSeq is platform independent and has been tested in Windows, Mac and Linux systems.

VoSeq consists of two interfaces, an Administrative interface for uploading and updating data, creation of user accounts, taxon sets and gene descriptions, and a User interface for data query and retrieval.

**Figure 1:**
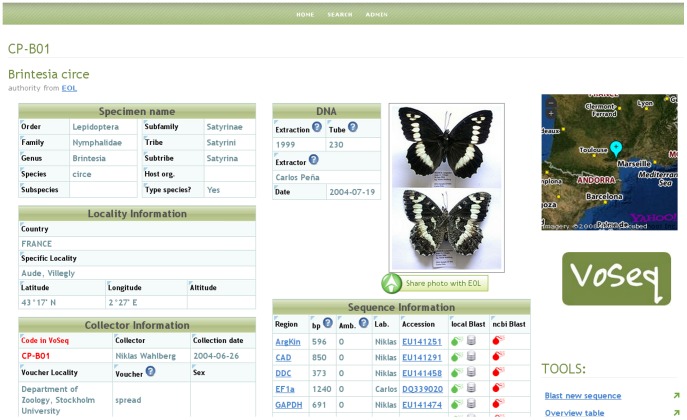
Screenshot of voucher page in VoSeq. It shows specimen and collection data, links to DNA sequences in GenBank, local and remote BLAST tools and mash-ups with Flickr and Yahoo Maps! for voucher picture and geographic location.

In the User interface, all available data for each voucher sample is summarized in its voucher page. When available, this page (Figure 1) shows taxonomic data, collection information along with an interactive map, a voucher picture and the list of DNA sequences including gene region, number of base pairs, accession numbers and a link to a “sequence page” where users can access the actual sequence and other information such as primers used in PCR amplification. Vouchers can be searched through an advanced search tool. Queries are made by searching either single or combinations of fields. Most of the search fields, as well as other entry fields within the database, are fitted with auto-complete drop-boxes for user-friendliness (based on Ajax).

The Administrative interface allows users to create new entries, upload and update all data and post voucher pictures that are hosted in the web service Flickr [11]. When users upload a picture in VoSeq, an algorithm will post the picture to Flickr and register the web addresses in MySQL. This facilitates showing the picture in the corresponding voucher page. This Flickr plug-in is enabled by following the instructions that will appear in VoSeq the first time a user tries to upload a picture. Once the user obtains an account in Flickr, VoSeq will instruct how to get a private key and how to register it in VoSeq's configuration file (this process needs to be done only once). New voucher and sequence records can be created in VoSeq by using the Administrative interface for single entries. Voucher codes in VoSeq are unique and cannot be overwritten, but can be changed. Moreover, VoSeq also includes a tool for uploading batches of voucher data and sequences. Data from a MS Excel sheet or other tab-delimited table can be copied and pasted into VoSeq and all the data will be processed and stored ready to be queried and retrieved. Records that are being used in phylogenetic projects can be included into “taxon-sets”, either manually by marking the voucher in an overview table or added as a list, in the Administrative interface. Taxon-sets allow easy querying of voucher and sequence information or creation of datasets to be analyzed in phylogenetic software.

VoSeq is password protected and at installation open only to the installer, but additional user accounts are easily created, with or without administrative rights.

### Data retrieval

One key feature of VoSeq is the possibility to harvest batches of DNA sequences for phylogenetic analysis. Users can create datasets consisting of selected DNA (or amino acid) sequences including flexible choices of outgroup and ingroup taxa, gene markers, codon positions and taxon-sets as well as different partitioning schemes. The chosen data are retrieved in ready-to-run datasets in NEXUS [12], TNT [13] and PHYLIP [14] file formats to be used, as-is or modified, as input to phylogenetic software such as MrBayes [15], TNT, RAxML [16] and others.

VoSeq also includes BLAST capabilities [17]. By following on-screen instructions, users can install NCBI BLAST software used for finding matching homologous sequences, for a new sequence or an already stored sequence, among those hosted in VoSeq. It is also possible to BLAST users' sequences against those hosted in GenBank.

VoSeq also includes a tool to create MS Excel tables with information on voucher specimens and available sequences, as well as accession numbers for sequences used for preparing a manuscript for publication. These tables are practically ready to be included in submission to journals. FASTA files appropriate for submission of chosen taxa and sequences to GenBank are also readily made with a few mouse-clicks. These fields include information such as organism name and lineage, gene codes and specimen voucher codes and can be imported into software such as Sequin [18].

### Relation to other databases

In addition to BLAST capabilities against sequences in GenBank, VoSeq facilitates integration with other biological databases and public services available on the web. VoSeq is able to create a mash-up using Yahoo! Maps to plot the collection locality of vouchers. If the database has geographic coordinates for the particular voucher, its page will show the map with a tag pinpointing the specific locality for the voucher. This map can be zoomed in and out and dragged around by using the mouse. This is accomplished by implementing the Yahoo! Maps web service [19].

When users upload a voucher photo to VoSeq, it is automatically hosted in the user's Flickr account. From the voucher pages in VoSeq, by clicking the “Share with EOL” button, VoSeq will automatically submit voucher photos to the photo pool of the Encyclopedia of Life (EOL) [20] that is also hosted in Flickr.

VoSeq makes automated calls to EOL's web services [21] in order to pull information on authors and year of description for species. VoSeq sends genus and species names and waits for a response. If EOL response is positive, the full species name will be included in voucher pages.

VoSeq also facilitates sharing data with GBIF [22] by creating single-click data dumps that can be processed with their Integrated Publishing Toolkit (IPT), which is the method preferred by GBIF.

A very detailed error reporting system is used for minimizing downtime and making VoSeq as user-friendly as possible.

### Availability and future directions

The software is open source with a GPL v2 license: https://github.com/carlosp420/VoSeq. A test installation with sample data can be found at http://www.nymphalidae.net/VoSeq. The full documentation and how-to help can be accessed here: http://nymphalidae.utu.fi/cpena/VoSeq_docu.html.

VoSeq is a useful web-based database application aimed for molecular systematics. VoSeq's simple and intuitive interfaces allow users to organize molecular sequences and related biological data. Its tools for retrieving batches of sequences in FASTA format allows them to be easily exported to specialized software, while the tool for creating PHYLIP, NEXUS and TNT ready-to-use datasets prove very time-saving.

VoSeq is entirely open source (available on Github at [23]) and savvy users can tweak the code to produce variants for subsets of data, as well as adding specific extra functions. Github is a convenient platform for sharing VoSeq's source code because it can be easily branched into new projects. For example, the source code could be made to call external programs for automatic analysis runs in wanted applications, or users can add extra fields to the database suitable to their organism group and research project, such as additional environmental variables or behavioral information. We are also planning to incorporate a fast Neighbor-Joining algorithm to the dataset section for a quick and easy look at hypothesized relationships among chosen taxa/taxon set. We do like to keep the software focused on the main functions, e.g. voucher and sequence data storage and retrieval, but users are free and welcome to suggest additions to or modification of the application.

As the Internet is currently the most important medium for delivery of data, biodiversity informatics needs tools that automate the exchange of data over the Internet in order to integrate biological information available from a disparate array of sources [24]. One way of solving this problem is by having loosely interconnected databases over the Internet so that all relevant data regarding species of interest can be aggregated on-the-fly and be presented to users in only one website (e.g. [25]). Thus, all digitized biological information will be readily available to people. All that is needed is (1) tweaking existent and new databases to provide data in format that computers understand (i.e. XML, JSON and variants) and (2) use of unique identifiers [26].
